# Commensal communication to the brain: pathways and behavioral consequences

**DOI:** 10.3402/mehd.v23i0.19007

**Published:** 2012-08-24

**Authors:** John Bienenstock

**Affiliations:** 1 Department of Pathology and Molecular Medicine, McMaster University, Hamilton, ON, Canada; 2 Department of Medicine, The McMaster Brain-Body Institute, St. Joseph's Healthcare, Hamilton, ON, Canada

**Keywords:** probiotics, enteric nervous system, behaviour, autism, brain

In recent years there has been an explosion of investigation of the microbiota which constitutes the gut microbiome (1–5). The trillions of bacteria which together make this up are engaged in multiple interactions with each other and the host in which they live. Indeed, they have been regarded as an additional organ within the external compartment of the body which influences the endocrine, metabolic and immune functions of the host. It has only recently received attention from the point of view of influence on the nervous system and this aspect has received the title ‘microbiome-gut-brain axis’ (6). This brief paper highlights some of the recent findings which promote the functional importance of this communication between gut commensals and the enteric and central nervous systems and was part of a lecture given recently at a meeting at the Karolinska Institutet, focused on ‘The Gut and The Brain’, with focus on Autism Spectrum Disorder (ASD).

It is well appreciated in clinical medicine that patients with hepatic encephalopathy are best treated with non-absorbable wide spectrum antibiotics and laxatives thus confirming the relationship between overgrowth of certain normal conventional bacterial gut contents and brain dysfunction (7). The gut–brain-axis refers to a bi-directional communication pathway which is contributed to by the gut microbiome.

It is also recognized that the irritable bowel syndrome (IBS) is associated with psychiatric dysfunction and it is argued by some that this is a common co-morbidity and that the key factors linking these together are what is called dysbiosis, which refers to an imbalance of the normal gut commensal population (8). IBS has also been connected with increased psychosocial stress especially since this has particular effects on the composition of normal gut microbiota. In this regard it is important to note the landmark observations by Sudo et al. (9) who showed that germ-free (GF) mice had exaggerated hypothalamic–pituitary–adrenal (HPA) axis responses to acute stress and that these were normalized by conventionalization with normal feces. Additionally, they could be restored to normal adult levels by monoassociation with a probiotic bacteria, *Bifidobacterium infantis*, but only if these treatments occurred in early life. Thus the HPA axis in these animals was programmable and programmed since attempts to treat mice in adulthood failed to reset the HPA response in terms of the corticosterone pathway.

On the other hand, Gareau et al. (10) showed that ingestion of two Lactobacilli was able to attenuate HPA responses if given in adulthood and this apparent discrepant observation is not yet resolved.

Heijtz et al. (11) showed that the brains of GF mice differed neurochemically and functionally from conventionally housed animals and that again early conventionalization of the gut microbiome restored these differences to those seen in adult life, suggesting the importance of the composition of the gut microbiome to normal brain development. We have also studied GF animals and have shown that they intrinsically express anxiolytic behavior and have alterations in the neurotransmitter receptors for NMDA and 5-HT in specific brain areas and also have reduced brain-derived neurotophic factor (BDNF) expression in the dentate gyrus of the hippocampus which is compatible with their behavioral phenotype (12). Indeed, different strains of mice have different behavioral phenotypes and Bercik et al. (13) have shown remarkably that this seems to depend on the normal microbiome of each mouse strain since fecal transplantation of one strain's microbiome to another either in antibiotic treated or GF states, conferred the donor's behavior to the recipient.

From the observations listed above, questions clearly arise as to how non-pathogenic bacteria in the intestine communicate with the brain. This aspect of communication is still in its early days yet we are beginning to obtain clues as to possible mechanisms. The ingestion of a probiotic, *Lactobacillus rhamnosus* (JB-1) was shown to inhibit visceral pain responses in rats subjected to colo-rectal distension and this was accompanied by a decrease in electrical responses in the afferent fibers of the dorsal root ganglia (14). Similar inhibition of pain was seen in mice treated with non-absorbable antibiotics (15) or another strain of Lactobacillus and opioid and cannabinoid receptors were upregulated in gut epithelia exposed to yet another Lactobacillus in association with anti-nociception (16). These studies emphasize the fact that single commensal bacteria given to normal animals can change their visceral pain perception.

Several further pieces of evidence support these observations. Exposure of the intestinal lumen to *L. rhamnosus* (JB-1) in both rats and mice, has shown that a specific enteric nerve subtype, AH but not S cells are affected by this treatment in a uniform manner within minutes of application of bacteria into the intestinal lumen (17). The conclusions were that the bacterium directly or indirectly inhibits the calcium activated potassium channel in these cells and closely mimics the specific channel blocker, TRAM 34. Similar results obtain from experiments looking at patch clamped myenteric plexus AH neurons from animals which had ingested the same bacterium for several days (18). In none of these experiments did another Lactobacillus (*L. salivarius*) have these effects suggesting that the functional effects of such bacteria are highly strain specific.

We have examined the effects of 28 days oral treatment of mice with *L. rhamnosus* (JB-1) on behavior and shown it to have an anxiolytic effect at the same time altering the expression of GABA A and B receptors in specific parts of the brain in a manner compatible with a benzodiazepine-like effect (19). At the same time we found that this probiotic treatment also attenuated the HPA axis response to acute stress despite administration in adulthood. However, when the animals were subjected to sub-diaphragmatic vagotomy prior to ingestion of the probiotic organism, both behavioral and central neurochemical changes were abrogated.

From the data highlighted herein, it is reasonable to conclude that the balance of normal bacteria in the intestine can have far reaching effects on both brain development and function, even in adults. The mechanisms and pathways which are utilized may be different in the case of different organisms and different genetic backgrounds since vagotomy does not always prevent behavioral changes. The extent to which these experimental results can be extrapolated to the human and found to be relevant in the clinical setting remains to be settled but the experimental evidence presented certainly emphasizes the potential importance of further detailed exploration of the principles beginning to be established in animals.
